# Implantoplasty-Aided Prosthetic Rehabilitation for the Management of Peri-Implantitis in Maxilla Reconstructed With Free Fibula Flap: A Clinical Report

**DOI:** 10.1155/crid/7502566

**Published:** 2025-01-16

**Authors:** Ajay Yerramsetti, Manju Vijayamohan, Maya Rajan Peter, Pramod Subhas

**Affiliations:** ^1^Amrita Institute of Medical Sciences and Research Centre, Amrita Vishwa Vidyapeetham, Kochi, India; ^2^Department of Prosthodontics, Amrita School of Dentistry, Amrita Vishwa Vidyapeetham, Kochi, India; ^3^Department of Periodontics, Amrita School of Dentistry, Amrita Vishwa Vidyapeetham, Kochi, India; ^4^Department of Cranio-Maxillofacial Surgery, Amrita Institute of Medical Sciences and Research Centre, Amrita Vishwa Vidyapeetham, Kochi, India

**Keywords:** free fibula flap, implantoplasty, peri-implantitis, prosthetic rehabilitation

## Abstract

For managing peri-implantitis, a variety of treatment modalities involving both surgical and nonsurgical methods including implantoplasty have been proposed. Implants that are placed in a free fibula flap are more prone to peri-implantitis due to the absence of firm, keratinized mucosa. Prosthetic design that offers adequate hygiene access should be designed whenever possible; otherwise, it may lead to the accumulation of plaque or biofilm that may lead to peri-implant diseases. Implantoplasty, which is performed in advanced peri-implantitis cases that cause exposure of implant threads, has been proposed as a reliable approach to preventing progressive peri-implant bone loss by modifying the exposed implant surface. This clinical report details the management of severe peri-implant bone loss in a maxilla reconstructed with a free fibula flap by combining soft tissue management, implantoplasty, and prosthetic rehabilitation.

## 1. Introduction

The occurrence of various biological complications might reduce the life span of dental implants. Peri-implantitis, which is regarded as a biofilm-mediated inflammatory condition, is regarded as the main reason for implant failure [1]. It is defined as an inflammatory reaction associated with the loss of supporting bone beyond initial biological bone remodeling around an implant in function. It is diagnosed based on bleeding on probing with radiographic signs of bone loss and attachment. It is also accompanied by suppuration and increased depth on probing [2, 3].

According to the morphology of defects, peri-implantitis is of three types [4, 5]:

• Type I—infraosseous/vertical defect
• Type II—supracrestal/horizontal defect
• Type III—combined defect

In general, various factors like soft tissue–implant interface, smoking, systemic diseases, and plaque control have appeared to be associated with peri-implantitis [6]. Local predisposing factors for the onset and advancement of peri-implantitis include the design of the prosthesis. The prosthesis has to be both biologically and functionally balanced in terms of providing adequate cleansable area along with strength, phonetics, and aesthetics [7].

Microvascular free fibula flap reconstruction is considered the standard method of treatment for postsurgical defects in patients with neoplasm, trauma, osteonecrosis, and congenital defects of the oral cavity and oropharynx in both the mandible and maxilla. Free fibula flap provides adequate bone for the placement of endosseous implants that helps restore lost function and aesthetics [8, 9]. In maxillary reconstructions, common reasons for implant failures include insufficient prosthetic space, inappropriate maxillomandibular relationships, and mobile soft tissue [8].

The primary goals of peri-implantitis therapy are to resolve inflammation and to arrest the progression of the disease [2]. Various treatment options that are available to treat peri-implantitis include surgical, nonsurgical, regenerative, and resective methods, along with different surface decontamination approaches [10]. The resective procedure involving modification of the exposed implant threads into a smooth and polished surface to achieve good biological outcomes in stabilizing bone loss is called implantoplasty [11].

According to many authors, peri-implantitis accelerates more rapidly at rough implants when compared to machined surface implants [12]. Implantoplasty has been advised in combination with the surgical type of treatment for peri-implantitis. It involves modifying the exposed implant threads using a series of rotary instruments to provide a smooth surface by removing implant threads. This technique helps to provide a smooth implant surface that facilitates effective biofilm and plaque control, thereby enhancing the hygiene around the implants [10, 12–17].

As this procedure generates thermal changes, it must be carried out with an adequate coolant [18]. A few clinical studies reported successful outcomes when surgical procedures are combined with implantoplasty. According to Romeo et al., among the various treatment options available for peri-implantitis, surgical debridement combined with implantoplasty has better outcomes [6]. According to Romeo et al., Pommer et al., Matarasso et al., and Schwarz et al., implantoplasty procedure provided short-to-medium-term enhanced clinical and radiographic results like low bleeding rates, reduced pocket depths, improved clinical attachment levels, and stable bone levels [6, 18–20]. Its limitations include damage to the implant body and implant-abutment connection, overheating of the surrounding bone, mucosal staining, and inflammatory reactions from the titanium particles generated during the procedure [12, 15]. This article describes a comprehensive treatment procedure for treating peri-implantitis in a maxilla reconstructed with a microvascular free fibula flap, combining implantoplasty with optimal prosthetic rehabilitation.

### 1.1. Clinical Report

A 45-year-old man was reported to the Department of Maxillofacial Prosthodontics with a chief complaint of a broken upper denture and an exposed metal plate in the upper left back region. His medical history revealed that he underwent bilateral maxillectomy due to rhino-orbital mucormycosis and was later reconstructed with an osteocutaneous free flap. Prosthetic rehabilitation was done using six dental implants.

**Figure 1:**
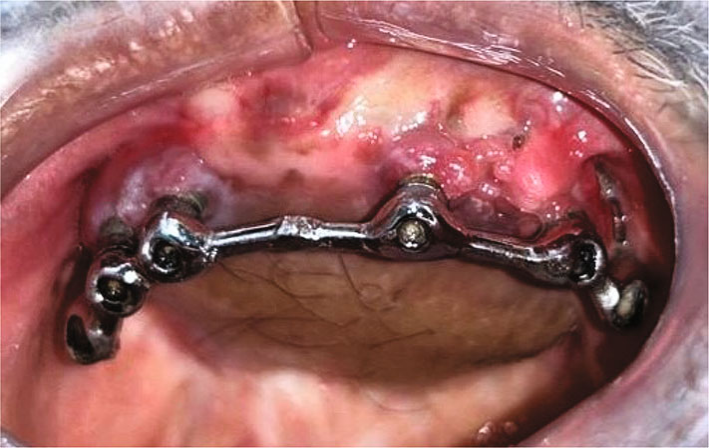
Soft tissue proliferation and exposed reconstruction plate.

**Figure 2:**
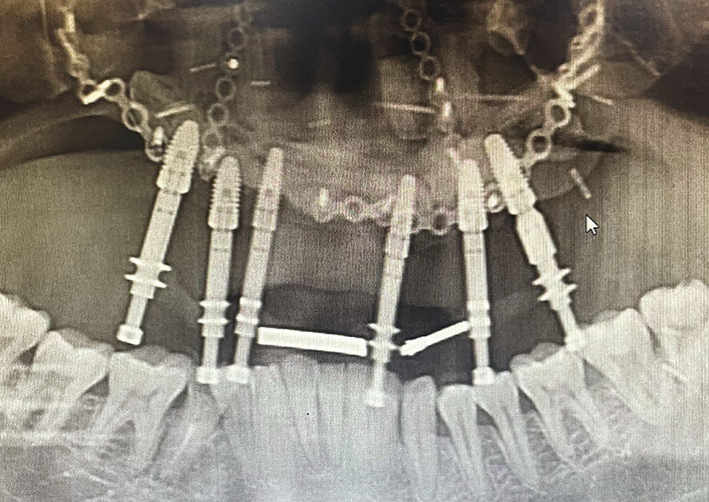
Orthopantomography showing marked bone loss.

On intraoral clinical examination, there were six implants (13 and 23 [area 4.3∗11.5 mm]; 14 and 16 [area 5∗10 mm]; 25 [area 5∗10 mm]; and 26 [area 5∗11.5 mm] (NobelReplace/Select/Conical/Connection, Nobel Biocare)) splinted with a bar, all the six implant threads were exposed (≥ 5 mm), hyperplastic soft tissue around the implants and exposed reconstruction plate on the left buccal area (Figure 1). An orthopantomography was done to evaluate the extent of bone loss which revealed marked bone loss (Figure 2). The patient mentioned that he is not willing to undergo any other additional major surgical procedure and also desires a new prosthesis in a brief period.

### 1.2. Treatment Plan

Considering the existing intraoral condition and keeping patient requirements in mind, a multidisciplinary team meeting was held. The primary goal is to cover the exposed reconstruction plate and remove the excess soft tissue and peri-implant submucosal curettage. Once the tissues are healed, implantoplasty and surface decontamination around the implants are scheduled. Simultaneously, root canal treatment followed by crown preparation was also planned to correct the lower anterior occlusal plane and to fabricate a new implant-supported overdenture that allows adequate access for hygiene measures.

### 1.3. Course of the Treatment

A minimal surgical approach consisting of submucosal curettage around the peri-implant tissues to remove the granulation tissue, implantoplasty, and chemical decontamination of the exposed implant surfaces was performed.

## 2. Soft Tissue Management and Implantoplasty

**Figure 3:**
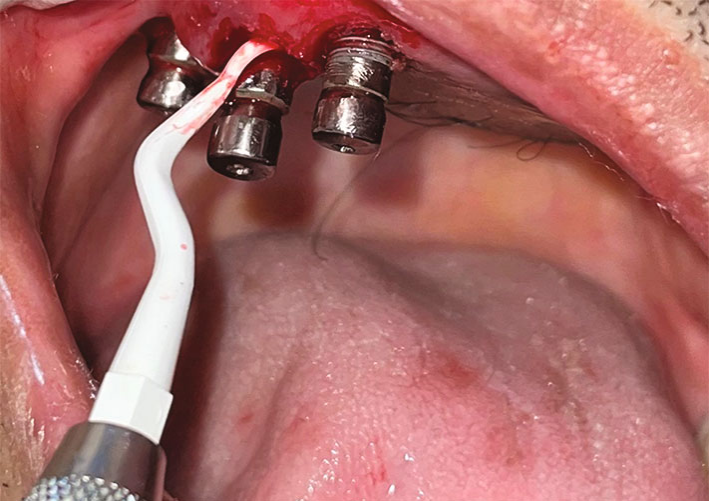
Granulation tissue removal using a plastic Gracey curette.

**Figure 4:**
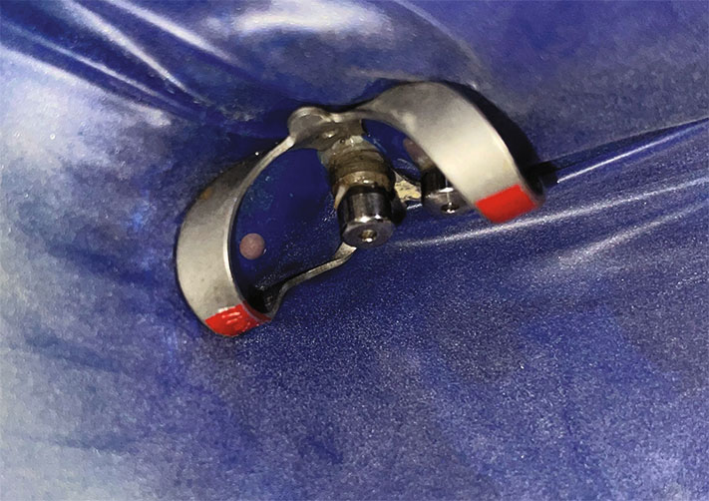
Isolation using a rubber dam to prevent titanium particle ingestion.

**Figure 5:**
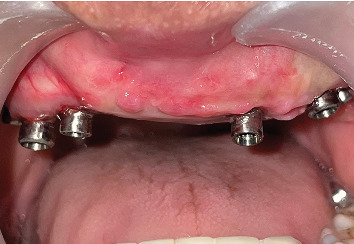
Well-healed soft tissue.

The patient was referred to the department of cranio-maxillofacial surgery to manage the exposed reconstruction plate. After achieving the closure of the exposed reconstruction plate, excess peri-implant soft tissue was removed using a laser, and peri-implant curettage was done using a plastic Gracey curette (Figure 3). Exposed implant threads were grounded using diamond burs (Coltene Diatech Crown Preparation Kit, COLTENE) and silicon polishers (Brownie and Greenie, Shofu) assembled on a handpiece working at 2,00,000 rpm under 2.5 × magnification loupes with adequate illumination. A rubber dam was used for isolating the implants to prevent the aspiration of titanium particles into the oral cavity and surrounding tissues (Figure 4). A new set of burs was used for each implant, and healing abutments were inserted to protect the implant connection from titanium debris. Finally, the surface decontamination of the exposed implant surfaces was done topically using tetracycline for 5 min. Marked soft tissue health was achieved a week after all these procedures (Figure 5).

### 2.1. Prosthetic Rehabilitation

**Figure 6:**
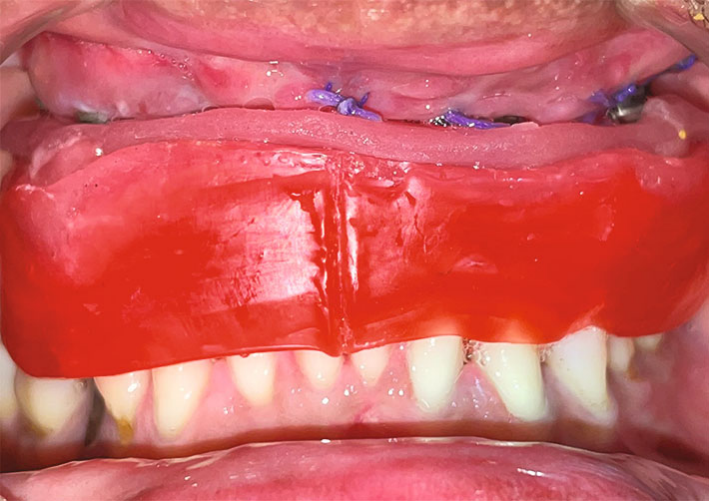
Jaw relation record after lower anterior teeth preparation.

**Figure 7:**
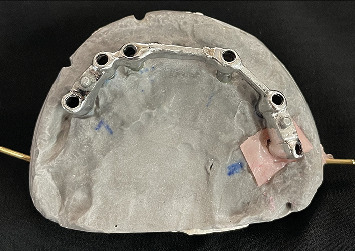
Splinted metal bar.

The aim of the prosthetic rehabilitation was to deliver a prosthesis that allowed good access to oral hygiene procedures. An open tray impression is made using putty light body material for the fabrication of bar-supported overdentures. Simultaneous endodontic treatment and tooth preparation were done to correct and align the lower anterior teeth, and later, jaw relation was recorded (Figure 6). A bar is fabricated with adequate soft tissue clearance for housing two sleeves anteriorly and ball attachments posteriorly on both sides to distribute the stresses evenly and to prevent any unwanted movement during function (Figure 7). During the try-in procedure, the maxillary denture was adjusted according to the aesthetics and lip fullness. Once the patient is satisfied with the aesthetics, lower temporary splinted crowns are adjusted accordingly and an index is made to communicate with the lab to replicate the exact contours achieved during the try-in procedure.

**Figure 8:**
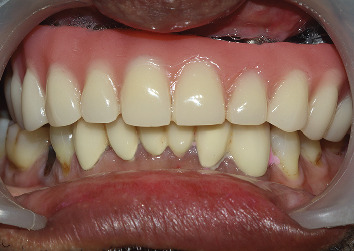
Finished prosthesis in occlusion.

On the day of final insertion, the bar is secured over the implants and torqued to 35 Ncm, and then lower permanent splinted crowns are secured using temporary cement. The final bar-supported overdenture is placed, and necessary occlusal corrections are made. Once the final occlusal corrections are made, the permanent splinted crowns are cemented using glass ionomer cement (Figure 8). A soft splint on the mandibular arch is delivered and advised the patient to wear it during sleep to prevent unwanted contact of the bar over the lower anterior crowns and remaining dentition. Adequate oral hygiene measures were instructed to the patient to maintain the exposed implant surfaces. Re-evaluation was carried out at 3- and 6-month intervals to assess probing depth, bleeding on probing, and the presence or absence of plaque.

**Figure 9:**
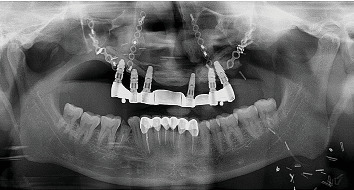
Orthopantomogram during the follow-up visit.

Reduced probing depth and stable bone levels were noted during the follow-up intervals (Figure 9).

## 3. Discussion

Peri-implant bone loss in the free fibula flap was found to be associated with hyperplastic tissue. This type of tissue results mostly due to the absence of peri-implant keratinized mucosa and difficulty in managing peri-implant hygiene [21]. Moreover, implant surfaces that are exposed to an oral environment with rough and free surface energy might markedly encourage bacterial colonization which will lead to peri-implantitis [16]. Treatment for peri-implantitis includes supragingival plaque removal, granulation tissue removal and decontamination of the exposed implant surface, correction of the bony defect, altering the exposed rough surface, and an effective plaque control regimen [6]. In cases with advanced peri-implantitis where bone loss is greater than 5 mm, implantoplasty is proven to be a clinically reliable procedure [7]. A combination of resective therapy along with implantoplasty has shown positive clinical results on implants affected with peri-implantitis [6]. Pommer et al. reported an 87.2% success rate in a 9-year follow-up retrospective analysis, and Romeo et al. reported an 87.5% collective implant survival rate in a 3-year follow-up randomized control trial for procedures involving implantoplasty [19, 22].

Controlling the deposition of titanium particles to the surrounding tissues is comparatively more challenging during implantoplasty, and variable amounts of deposition can be expected after the procedure [12]. These ions and particles that get released during corrosion or mechanical grinding of titanium implants have been shown to cause adverse allergic reactions in humans. Copious irrigation combined with suction has been suggested to minimize the deposition of titanium particles during the implantoplasty procedure [23].

Under given clinical conditions, the brownie and greenie silicone polishers are necessary to achieve the smoothest possible surface. Clinicians should also consider the contamination of the peri-implant site with silicone debris while polishing with these burs [16]. Minimal thermal changes ranging from 1.5° to 1.8° are noted under appropriate cooling conditions which pose no considerable thermal risk for the neighboring tissues. Moreover, most of the mentioned complications can be avoided if proper care is taken [12].

Sharon et al. studied the amount of heat generated by three different burs used in this procedure and concluded that under proper cooling conditions, implantoplasty did not generate a significant temperature rise that could be clinically relevant [15, 17]. According to Chan et al., the mean bending strength of narrow-diameter implants is greatly decreased by implantoplasty. Therefore, extreme care should be taken while performing implantoplasty on narrow-diameter implants [24]. Regular clinical and radiographic evaluation is paramount to recognizing and preventing peri-implant pathology at an early stage for implants in a free fibula flap [9]. Further research involving different implant designs is required to evaluate the long-term prognosis of this method [15].

This case report describes how an implantoplasty is performed using a three-bur protocol along with occlusal modifications to achieve an optimal prosthetic result. The advantage of this approach includes removing the exposed implant threads to provide a smooth, cleansable surface, thereby limiting the biofilm/plaque accumulation. Sufficient prosthetic space is provided between the tissue and prosthesis to make it easily accessible for the patient to perform oral hygiene procedures. Titanium particle deposition, compromised strength of the implant as the diameter reduces during the procedure, and added expenses for the fabrication of a new prosthesis are the limitations of this procedure.

## Data Availability

Data is openly available in a public repository that issues datasets with DOIs.
